# Fgf10/Fgfr2b Signaling in Mammary Gland Development, Homeostasis, and Cancer

**DOI:** 10.3389/fcell.2020.00415

**Published:** 2020-06-26

**Authors:** Stefano Rivetti, Chaolei Chen, Chengshui Chen, Saverio Bellusci

**Affiliations:** ^1^Key Laboratory of Interventional Pulmonology of Zhejiang Province, Department of Pulmonary and Critical Care Medicine, First Affiliated Hospital of Wenzhou Medical University, Wenzhou, China; ^2^Cardio-Pulmonary Institute and Institute of Lung Health, Universities of Giessen and Marburg Lung Center, Member of the German Center for Lung Research, Justus-Liebig-University Giessen, Giessen, Germany

**Keywords:** Fgf10, Fgfr2b, mammary gland, development, stem cells, cancer

## Abstract

Fibroblast growth factor 10 (Fgf10) is a secreted ligand acting via the Fibroblast growth factor receptor 2b (Fgfr2b). Fgf10/Fgfr2b signaling plays important roles both in the epithelium and in the mesenchyme during mammary gland development. Evidence in mice show that Fgf10 is critical for the induction of four out of five of the mammary placodes and for the formation of the white adipose tissue. Fgfr2b ligands also play important function in the maintenance of the terminal end buds, specialized structures at the tip of the ramified ducts during the postnatal phase of mammary gland development. Finally, in humans, FGF10 has been described to be expressed in 10% of the breast adenocarcinoma and activation of FGFR2b signaling correlates with a worse prognostic. Therefore, Fgf10 plays pleiotropic roles in both mammary gland development, homeostasis and cancer and elucidating its mechanism of action and cellular targets will be crucial to either enhance mammary gland development or to find innovative targets to treat aggressive breast cancer.

This review article focuses on the role of Fibroblast growth factor 10 (Fgf10) both pre- and post-mammary gland formation. We provide evidence that this dual role is conserved in the lung and limb, where Fgf10 has been shown to be important. In addition, we propose that the molecular mechanisms regulating embryogenesis are conserved during post-natal development as well as in breast cancer progression.

## Fgf10 Belongs to the Fgf7 Subfamily of Secreted Growth Factors Acting Mostly Through Fgfr2b

The Fibroblast growth factor 7 subfamily is made of four secreted growth factors (Fgf3, Fgf7, Fgf10, and Fgf22). Initial studies took advantage of a conserved region between the first two members of this family (Fgf3 and Fgf7) to identify additional subfamily members (Fgf10 and Fgf22). In addition to sequence identity, the members of this subfamily also bind to common receptors albeit with different affinities (Ornitz and Itoh, 2001). Fgf10 in particular plays a crucial role during organogenesis. Fgf10, which is mostly expressed by mesenchymal cells, acts principally through the Fgf receptor 2b (Fgfr2b) and Fgfr1b expressed in the epithelium to control the formation of ramified structures such as the embryonic lung (Bellusci et al., 1997). Fgf10 elicits its action through chemotaxis, which involves a coordinated migration of an epithelial sheet toward a localized source of Fgf10 (Park et al., 1998; Weaver et al., 2000; Jones et al., 2018, 2019). Consistently, *Fgf10* or *Fgfr2b* null mice display agenesis of many organs such as the lung, limb and mammary gland (Min et al., 1998; Sekine et al., 1999; Ohuchi et al., 2000; Mailleux et al., 2002).

## Early Mammary Gland Development Starts With the Formation of Ectodermal-Derived Placodes

Mammary gland formation in the mouse begins around embryonic day 10 (E10) with the formation of two mammary lines, located in antero-posterior direction along each flank of the embryo (Turner and Gomez, 1933). By E11-E12, five lens-shaped structures, the mammary placodes, are detected along each mammary line, as ectodermal thickenings that in 24 h develop into epithelial buds. These buds, three thoracic and two inguinal, are located at reproducibly precise positions. This reproducibility suggests a tight spatial-temporal control of placode induction. However, the genes involved in such regulation are still unclear. Previous reports suggest that mammary placodes in rabbit are formed by the migration of ectodermal cells along a mammary line (Propper, 1978), rather than by local increase in cell proliferation (Balinsky, 1950). In agreement with these results, analysis of proliferation in mice indicated that cells that contribute to the mammary placode are proliferating less than the adjacent cells in the surface ectoderm (Lee et al., 2011). In addition to cell migration, it was also shown that these placode cells undergo hypertrophy (Lee et al., 2011). Therefore, both cell migration and cell hypertrophy contribute to the growth of the mammary placode, regardless of their thoracic or inguinal position.

## *Fgf10* and *Fgfr2b* Null Mice Fail to Develop Normal Mammary Glands

Little is known about the genes that regulate the induction of the mammary placodes and the early phases of mammary gland development. However, there are several indications for the requirement of Fgf10-signaling via Fgfr2b for placode induction and development. It was previously described that the formation of the mammary line as well as the subsequent induction of four out of five mammary placodes was impaired in both *Fgf10* and *Fgfr2b* knock out embryos (Mailleux et al., 2002). Interestingly, in the *Fgfr2b* KO, the mammary placode number 4, situated inguinally progressively disappeared between E11.5 and E13.5 through decreased proliferation and increased apoptosis of the mutant epithelium. In the *Fgf10* KO, the mammary bud 4 is still present due likely to the redundant expression of Fgf7, another ligand of Fgfr2b (Mailleux et al., 2002). It has been therefore proposed that Fgfr2b signaling is important to control survival and proliferation of the mammary epithelium during the branching morphogenesis phase of mammary gland development. Interestingly, the expression of Fgfr2 is elevated after weaning and remains high in virgin mice only to decrease during pregnancy and lactation. This increase during the weaning period and in the virgin stage can be directly associated with the tremendous ramification process taking place during that time. Interestingly, compared to Fgf7 expression, Fgf10 expression is expressed 15 times higher (Pedchenko and Imagawa, 2000) suggesting that Fgf10 takes also center stage during the postnatal phases of mammary gland development.

## A Possible Interplay Between Fgf and Wnt Signaling is at Work for Mammary Placode Formation

Wnt signaling is likely also connected to Fgf10 signaling during mammary placode development as demonstrated by a mammary gland agenesis phenotype when Wnt signaling is inhibited (Andl et al., 2002). Lef1 (a transcriptional effector of Wnt signaling) is a well-described marker for mammary placode formation (Mailleux et al., 2002), and its ablation leads to an arrest in the bud phase (van Genderen et al., 1994). Moreover, transgenic overexpression of Dkk1, a secreted inhibitor of Wnt ligands leads to an arrest in mammary gland development prior to the bud stage (Andl et al., 2002).

**Figure 1:**
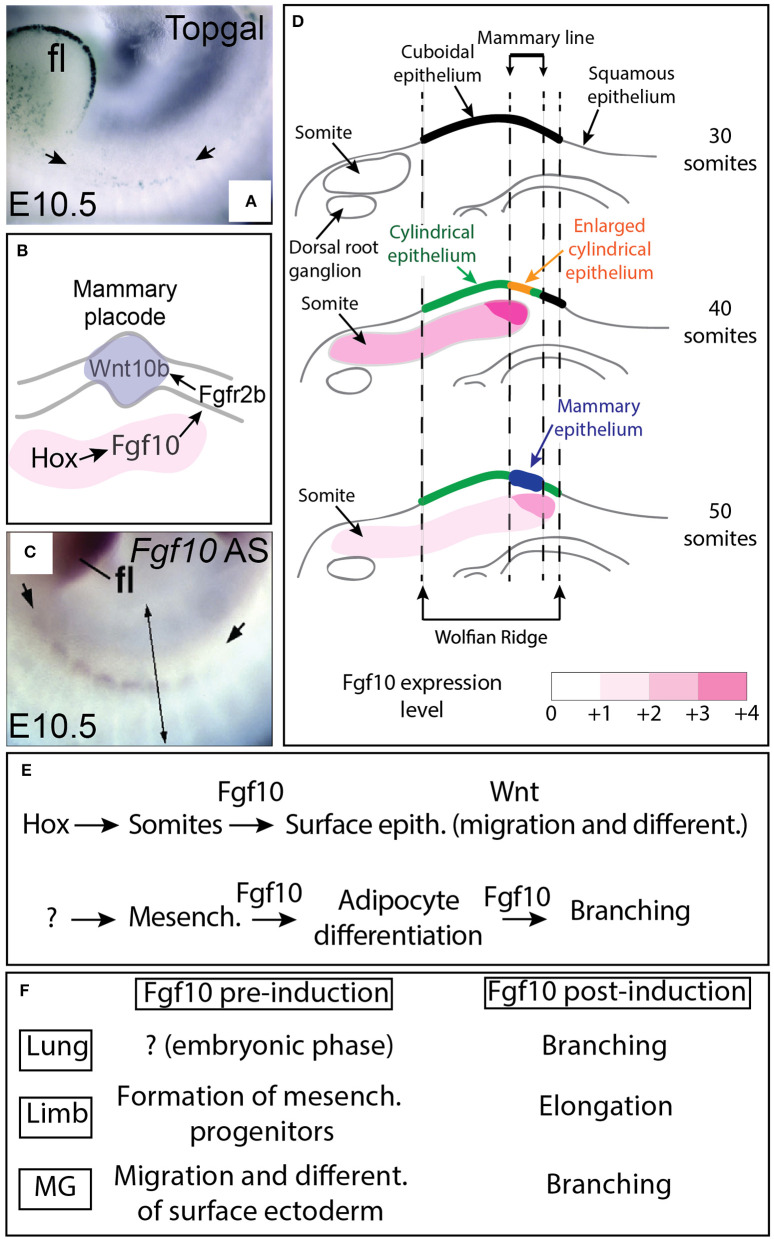
Fgf10 expression from the somites controls placode formation from the ectodermally-derived mammary line. **(A)** β-galactosidase expression in the Wnt reporter Topgal mice at E10.5 showing Wnt-responding cells in the ectoderm where the mammary line is forming (from Veltmaat et al., 2006). **(B)** Schematic model. Fgf10 expression in the somites is regulated by *Hox* genes, Fgf10 acts via Fgfr2b expressed in the ectoderm to trigger Wnt10b expression leading to Wnt signaling activation in the epithelium of the forming mammary line/placode. **(C)** *Fgf10* mRNA expression by *in situ* hybridization at E10.5 showing *Fgf10* expression in the somites (from Veltmaat et al., 2006). **(D)** Progressive differentiation of the ectoderm-derived epithelium in the mammary line from cuboidal (30 somites stage) to cylindrical/enlarged cylindrical (40 somites stage) to form the mammary epithelium constituting the placode (at 50 somites stage) is associated with increased Fgf10 expression as the somites grow dorsally. **(E)** Summary of Fgf10 regulation and activity during embryonic mammary gland development. *Hox* genes control Fgf10 expression in somites. Fgf10 from the somites acts on the surface ectoderm to trigger migration and differentiation. Fgf10 is also induced in the fat pad precursor through still to be identified mechanisms and acts on both the epithelium (to control branching) and the mesenchyme (to control adipocyte differentiation). **(F)** Fgf10 activity pre and post-organ induction in lung, limb and mammary gland. Scale **(A,B)**: 70 μm. fl: forelimbs. Simple arrows in **(A)** indicate the forming mammary line. Double arrow in **(C)** indicates a virtual section through the somites displayed in **(D)**.

Using the Topgal reporter, a mouse allowing to monitor the activation of β-catenin signaling via the expression of β-galactosidase (DasGupta and Fuchs, 1999), it has been shown that in the embryo at E10.5, the activation of the Wnt signaling could be detected in the ectoderm at the level of the putative mammary line (Figure 1A) (Veltmaat et al., 2006). In addition, Fgf10/Fgfr2b signaling controls the expression of several Wnt ligands including Wnt10b (Figure 1B) (Veltmaat et al., 2006). These results suggest that the Wnt pathway intersects with the Fgf10 pathway to control mammary placode formation (Veltmaat et al., 2003, 2004, 2006).

The answer to the question about how could Fgf10 and Wnt signaling intersect came from the analysis of the role of Fgf10 at early developmental stage of the mouse lung, another related organ which ramifies. This study was carried out using a double transgenic mouse allowing the doxycycline-based expression of a soluble form of Fgfr2b acting as a scavenger for all Fgfr2b ligands (Parsa et al., 2008, 2010; Jones et al., 2018). Unlike the genetic inactivation using the Cre/LoxP technology of *Fgr2b* or its ligands (which is even more challenging because of the potential redundancy between members of Fgf7 subfamily), this approach allows inhibiting efficiently Fgfr2b signaling within 1 h following intra-peritoneal injection of doxycycline to the pregnant females (Danopoulos et al., 2013). Fgf10 is the main Fgfr2b ligand expressed in the lung at early developmental stages. Fgf10 is normally expressed in the distal mesenchyme adjacent to the epithelial tips of the lung buds (Bellusci et al., 1997). These distal epithelial structures respond to Fgf10 signaling. This is illustrated by the expression at the epithelial tip of several Fgf10-downstream target genes such as *Etv4, Etv5, Shh*, and *Sox9* (Jones et al., 2018). In addition, the lung tip epithelium displays significant expression of phosphorylated (Ser-552) ß-catenin. This protein corresponds to a transcriptionally active form of ß-catenin thereby indicating Wnt signaling activation. The presence of phosphorylated (Ser-552) ß-catenin is drastically reduced in the lung tip epithelium following Fgfr2b signaling inhibition suggesting that in this cellular compartment, Fgf10 functions upstream of Wnt/ß-catenin signaling. IQ-1, a pharmacological inhibitor of the interaction between ß-catenin and P300 (Miyabayashi et al., 2007), was shown to reproduce most of the effects at the cellular and transcriptomic level induced by blockade of Fgf10 signaling (Jones et al., 2018). How is Fgf10 regulation of ß-catenin activity achieved is still unknown. One possibility is that decreased Fgf10 signaling impacts the stability of ß-catenin *per se* leading to a decrease in the cytoplasmic level of free ß-catenin. Such regulation can be the consequence of altered phosphorylation of ß-catenin leading to its degradation in combination with decreased availability of free ß-catenin in the cytoplasm. This last aspect could be achieved through the perturbation of cell adhesion. As ß-catenin associates with the cell adhesion molecule E-Cadherin (Cdh1), such interaction regulates the level of ß-catenin available for signaling. Interestingly, catenin delta 2 (Ctnnd2) has been reported to be a downstream target of Fgf10 (Jones et al., 2018). Ctnnd2 plays a functional role in cell adhesion by destabilizing Cdh1 (Lu et al., 1999; Kim et al., 2012). In the context of active Fgf10 signaling, such destabilization could therefore be associated not only with increased cell mobility (which is one of the characteristic of the cells responding to Fgf10 signaling) but also with increased availability of free ß-catenin (arising from the pool of ß-catenin initially associated with Cdh1) in the cytoplasm that can be used for signaling upon translocation to the nucleus. In the context of blockade of Fgf10 activity, the decrease in *Ctnnd2* expression is associated with increased Cdh1 expression and reduced presence of the transcriptionally active form of ß-catenin (Jones et al., 2018).

Interestingly, a similar observation was made in the in the context of limb bud development in a structure called the apical ectoderm ridge (AER). The AER is a transient epithelial structure located at the apex of the developing limb bud (between E9.5 and E13) and involved in the elongation of the limb bud. The functionality of the epithelial cells in the AER is dependent upon the maintenance of a Fgf10/ß-catenin signaling axis (for a review on this topic see Jin et al., 2018). A very significant decrease of ß-catenin in the AER is observed as early as 1 h after blockade of Fgfr2b signaling at E11. In addition, 6 h following the inhibition, it was found that the subcellular localization of ß-catenin in the AER was abnormal and consistent with the lack of recovery of Wnt signaling in this critical epithelial structure (Danopoulos et al., 2013). Considering how quickly both the free pool of ß-catenin in the cytoplasm and the transcriptionally active form in the nucleus are affected, it is very likely that post-translational regulations, in particular phosphorylation events, are involved. Advanced phosphoproteomic studies combined with the use of transgenic tools allowing the rapid and reversible inhibition of Fgfr2b ligands activity will shed new lights on this critical interaction between Fgf10 and ß-catenin. Equally important will be the further analysis of the role of Ctnnd2 and more generally cell adhesion to mediate the interaction between Fgf10 and Wnt/ß-catenin signaling during mammary gland formation.

## *Fgf10* Expression in the Somites Controls Mammary Placode Induction

The characterization of the expression of *Fgf10* and *Fgfr2b* during early embryonic mammogenesis has been instrumental to fully understand the nature of the epithelial-mesenchymal interactions orchestrating mammary placode induction as well as the mammary phenotypes displayed by the *Fgf10* and *Fgfr2b* null mutants. As epithelial cell migration is suggested to underlie placode formation, it was expected to find *Fgfr2b* expression in the surface epithelium located in the region from which the placode will be forming and *Fgf10* in the underlying dermal mesenchyme. While *Fgfr2b* is spatio-temporally expressed according to the expectation, surprisingly, *Fgf10* is not expressed in the region of the mammary line nor in the underlying dermal mesenchyme until E15.5 (Mailleux et al., 2002). However, *Fgf10* is expressed in the nearby dermamyotome of the somites at E10.5. It has therefore been proposed that *Fgf10* expression in this structure is involved in mammary placode induction (see Figure 1C). In a follow up study (Veltmaat et al., 2006), it has been reported that the somites underlying the inguinal placodes 2 and 3 expressed *Fgf10*, in a gradient-like pattern across and within these somites. It was therefore therefore proposed that Fgf10 expression in the dermamyotome is required for inguinal mammary placode formation and that the Fgf10 and the Wnt pathway cooperate to control cell migration during mammary placode induction. Several mutants displaying abnormal *Fgf10* expression in the somites were monitored for the status of Wnt signaling as well as cellular changes at the level of the ectoderm as read out of both the mammary line and mammary placode formation. In *Pax3*^*ILZ*/*ILZ*^ mutants (Relaix et al., 2003), characterized by the absence of ventral somitic buds, the position of the mammary line is relocated dorsally and is associated with absent placode 3. Similarly, in *Gli3*^*Xt*−*J*/*Xt*−*J*^ mutants [(Maynard et al., 2002), displaying a shortened somatic Fgf10 gradient dorsally] and in hypomorphic *Fgf10* mutants [(Kelly et al., 2001), characterized by a general decrease in Fgf10 expression], both the mammary line and the placode 3 are absent. Interestingly, exogenously applied beads soaked with recombinant Fgf10 grafted on the flank of mutant embryos, at the location of the somites where placode 3 develops, was sufficient to rescue the formation of placode 3 in both *Fgf10* null and *Gli3*^*Xt*−*J*/*Xt*−*J*^ mutants. It is tempting to speculate that the progressive increase in somitic Fgf10 expression is causative for the progressive maturation of the surface ectoderm (Figure 1D). The current model proposes that Fgf10 expression in the somite, downstream of the transcription factor Gli3, is critical for the flank ectoderm to be committed toward the mammary epithelial lineage. Fgf10 gradients pattern across and within the somites, associated with the spreading of the somites ventrally, are instructive for the correct spatial positioning of the committed mammary epithelium. Interestingly, Homeobox (*Hox)* genes have been proposed to also play a critical function in the induction of the mammary placodes. In particular, *Hoxc8* expression is significantly detected at E10.5 in the surface ectoderm where the mammary line is forming and its ectopic expression using the *Wnt6-Cre* driver line both in the surface ectoderm and in the somites in the thoracic region leads to the formation of ectopic mammary placodes which were positive for *Wnt10b*. Interestingly, *Fgf10* expression was also increased in the thoracic somites (Carroll and Capecchi, 2015). It is therefore proposed that *Hox* genes both in the somites and in the surface ectoderm are important for mammary placode induction (Figures 1B,E).

It is important to mention at this point that Fgf10/Fgfr2b signaling is important not only for the induction of the mammary placode (Figure 1F) but also plays important function at later developmental stages during the process of branching of the mammary epithelial tree (see thereafter the chapter about the formation of the mammary epithelial tree). Interestingly, Fgf10 plays also multiple roles in the formation of the limb, which is another ectoderm-derived organ. Understanding the role of Fgf signaling in the limb is relevant for the understanding of mammary gland biology as the basic cellular and molecular mechanisms involved in the induction and the subsequent formation (elongation for the limb and branching for the mammary gland) of these 2 organs are likely conserved (see Figure 1F). Fgf10/Fgfr2b signaling was initially reported to be important in limb formation for the induction of the apical etodermal ridge (AER) and for the elongation of the limb bud along the proximal distal axis (Danopoulos et al., 2013; Jin et al., 2018). These two processes (AER induction and limb elongation) could be compared to the process of mammary placode formation and the subsequent bud formation, elongation, and branching. Interestingly, in the limb, Fgf10 has been reported to play a key role before the induction of the AER which takes places at embryonic day 9.5 and E10 for the forelimb and hindlimb, respectively. Before AER induction, Fgf10 has been proposed to control the process of epithelial to mesenchymal transition for the somatopleural epithelium. Such transition is instrumental for the generation of limb progenitors (Gros and Tabin, 2014). Fgf10 plays therefore important roles both before the induction of the AER (which was thought to be the earliest event in limb formation) as well as before mammary placode induction. It is not clear if this dual function played by Fgf10 prior and after organ induction is also conserved for other branched structures either endoderm-derived (lung, pancreas, cecum) or ectoderm-derived (teeth, tongue, palatal shelves as well as salivary and lacrimal glands for example) [For a comprehensive review on the role of Fgf10 in cranio-facial development see (Prochazkova et al., 2018)]. Interestingly, in both endoderm [especially the lung, see (Jones et al., 2018)] and ectoderm-derived organs (particularly for the cleft palate), a common feature appears to be the role of Fgf10 signaling in modulating cell adhesion. It is also clear, however, that Fgf10 signaling plays an important role in cell differentiation (Veltmaat et al., 2006; Jones et al., 2018).

## The Epithelium of the Mammary Gland 4 in *Fgf10* KO Embryos did not Undergo Branching Morphogenesis- A Phenotype Caused by Defective Fat Pad Formation

Branching morphogenesis in mammary gland development starts during embryogenesis but occurs mostly postnatally. Around E15.5, the epithelial buds elongate to invade the fat pad progenitor and subsequently ramifies at E16. At birth, the mammary rudiment displays main and accessory ramifications (Lyons, 1958; Nandi, 1958; Sakakura, 1987; Watson and Khaled, 2008). Analysis at E18.5 of the *Fgf10* KO mammary gland indicated that the mammary gland epithelium remained as a sprout failing to ramify. Close up examination of the gland indicated a very thin, underdeveloped mammary fat pad. Transplantation experiments of the mutant mammary gland epithelium from the *Fgf10* KO embryos into a cleared fat pad from wild type mice indicated that the mutant mammary epithelium was still capable of expanding and forming a complete mammary tree with well distinguishable terminal end buds similar to wild type mammary glands (Mailleux et al., 2002). These results suggested that lack of Fgf10 signaling in the mutant mammary epithelium did not lead to the loss of the stem cell capabilities of this rudimentary structure. In addition, the impaired fat pad in *Fgf10* KO mammary glands suggested that Fgf10 was important for the normal formation of the mammary fat pad, or more generally for the formation of the adipocytes. At this point, it is not clear if in addition of proper fat pad formation, Fgf10 itself is required for the mammary epithelial sprout invasion and ramification. This question is so far difficult to address during mammary gland development as Fgf10 plays a dual function. Fgf10 targets not only the epithelium, mostly via Fgfr2b but also the mesenchyme (likely via a combination of Fgfr2b and Fgfr1b, Al Alam et al., 2015) to trigger its differentiation toward the adipocyte lineage (Figure 1E). This conclusion about Fgf10's role in the mesenchyme, arising from the analysis of the mammary gland derived from placode 4 in *Fgf10* KO embryos was confirmed by an elegant study showing that Fgf10 acts directly on the mesenchyme via the transcription factor C/EBPß to control the differentiation of the pre-adipocytes into adipocytes (Sakaue et al., 2002). Interestingly, a similar situation is found during lung development where from E16.5 onwards, Fgf10 acts both on the alveolar epithelial progenitors to control their differentiation toward the alveolar epithelial type 2 lineage (Chao et al., 2017) as well as on the mesenchyme to control its differentiation toward the lipogenic lineage (Al Alam et al., 2015).

## Role of Fgfr2b Ligands in Terminal End Bud (TEB) Maintenance

**Figure 2:**
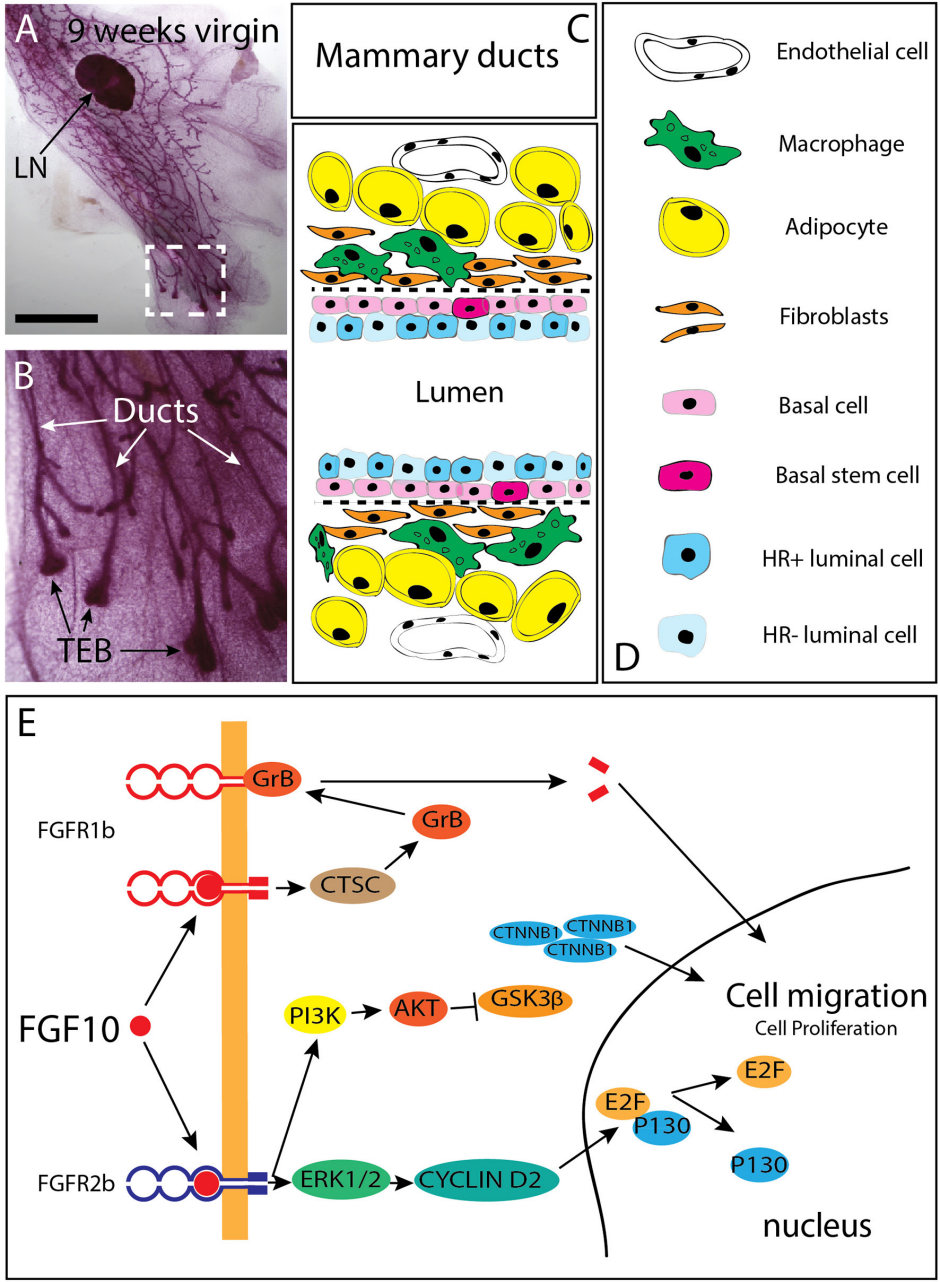
Branching of the mammary epithelial tree starts very early during fetal development and continues post-natally. **(A)** 9 weeks-old mammary gland from a virgin female mouse showing that the mammary epithelial tree has expanded quite distally compared to the lymph node (LN). **(B)** Magnification of the box shown in C. The mammary epithelial tree is made of terminal end buds (TEB) distally and ducts proximally. **(C,D)** Schematic of the mammary ducts in the adult mammary gland. Basal stem cells are located in the basal layer. These cells differentiate into hormone receptor (HR) positive and negative luminal epithelial cells as well as basal/myoepithelial cells. Mammary fibroblasts, which secrete many cytokines (including Fgf10) are located at close proximity of the basal layer. Fgf10 is also produced by adipocytes as it is needed for their differentiation. **(E)** FGF10 binding to FGFR1 leads to cleavage of the receptor by granzyme B and the translocation of a 55 kDa fragment of FGFR1 to the nucleus, leading to increased cell migration. FGF10/FGFR2b signaling complex activates extra-cellular signal-regulated kinases (ERK)/mitogen-activated protein kinases (MAPK) and phosphoinositide-3 kinase (PI3K). ERK kinases are responsible for the activation of cyclin D2 and consequent transcription of E2F targets genes. PI3K activates protein kinase AKT with subsequent inhibition of glycogen synthase kinase 3β (GSK3β) by phosphorylation, leading to an accumulation of beta Catenin (CTNNB1) and stimulation of the transcription of WNT-dependent genes MAPK dependent phosphorylation of transcription factors allows transcription of FGF target genes. CTSC, Cathepsin C; GrB, Granzyme B; PI3K, Phosphatidylinositide 3-kinase; ERK1/2, Extracellular signal-regulated kinase $\frac{1}{2}$; E2F, Transcription factor E2F; CTNNB1 Catenin beta 1; GSK3b, Glycogen synthase kinase 3 beta; AKT, Serine/threonine kinase. Scale **(C)**: 1.5 cm, **(D)** 100 μm.

During the 2 months-period following birth, the tips of the mammary rudiments keep invading the mammary fat pad and branching through a combined process of proliferation and differentiation (Hogg et al., 1983). At the cellular level, the mammary gland is made of ducts ending distally into a structure called “terminal end buds (TEBs) (Figures 2A,B). The ducts are composed of luminal epithelial cells which express or not the estrogen and progesterone receptor. Basal/myoepithelial cells are located around the luminal cells in the ducts (Figures 2C,D). Distally, the TEBs are dilated structures which appear around 3 weeks after birth as a result of hormonal changes (Williams and Daniel, 1983; Sakakura et al., 1987; Kouros-Mehr et al., 2006). Genetic deletion of the *estrogen receptor alpha* (*ER*α) leads to arrested mammary gland development postnatally with no terminal end buds forming (Feng et al., 2007). This phenotype is very similar to the one observed upon blockade of Fgfr2b signaling postnatally (Parsa et al., 2010) suggesting that Fgfr2b expression in the epithelium could be under the control of estrogen signaling. It is worth noticing that in the prostate, Fgfr2 expression is required for androgen activity and its expression is under the control of androgens (Lin et al., 2007; Yu et al., 2013). It is therefore possible that estrogens signaling, via the regulation of Fgfr2b expression in the epithelium, controls the response of the epithelium to the abundant presence of Fgf10 in the stroma.

The TEB structures contain mammary progenitors giving rise to both luminal epithelial and myoepithelial cells. In addition, bipotent progenitor cells located in the basal layer of the mammary duct (thereby called basal stem cells) represent another source of progenitors for the luminal epithelial and myoepithelial cells (Stingl et al., 2006; Rios et al., 2014). These basal stem cells present in the ducts are characterized by the expression of CD49f or CD29 (Shackleton et al., 2006; Stingl et al., 2006). The maintenance of the lumen structure within the mature mammary duct is important for efficient milk secretion (Streuli et al., 1991). Luminal epithelial cells are cuboidal cells tightly connected with each other and with myoepithelial cells. Loss in cell adhesion, and obstruction of lumen by tumor cells is commonly observed in earlier stages of breast cancer (Cardiff, 2010; Nistico et al., 2014).

Constant mutual interactions between mammary epithelial cells and their microenvironment mediated by paracrine signals control the behavior of the mammary epithelium (Fata et al., 2007; Alcaraz et al., 2008; Simian et al., 2009; Mori et al., 2013). Interestingly, TEBs disappear upon blockade of Fgfr2b signaling only to re-appear when Fgfr2b signaling is restored. A similar phenotype was observed for the incisors in mice indicating that the survival of the stem cells responsible for the formation/maintenance of the TEBs or the incisors is not dependent on Fgfr2b signaling (Parsa et al., 2008, 2010). While deletion of *Fgfr1* in the mammary epithelium using the *K14-Cre* driver leads only to a slight delay in development without any observable defect in mammary gland function, simultaneous ablation of both *Fgfr1* and *Fgfr2* (using an adenovirus-Cre approach) leads to a significant loss of self-renewal in the basal stem cell population (Pond et al., 2013). Transplantation experiments into a cleared fat pad of FACS-isolated basal stem cells showed that inactivation in Fgfr2 expression in these cells leads to a drastic reduction in self-renewal and impaired differentiation into luminal epithelial cells as well as defective epithelial branching (Zhang et al., 2014). In addition to an important role for Fgfr2b at the level of the TEB, Fgfr2b could also play an important role at the level of the ductal epithelium. In particular, the function of Fgfr2b signaling in the maintenance of cell adhesion in the mammary duct is still unclear. Our data recently published in the context of the embryonic lung clearly demonstrate that Fgf10/Fgfr2b signaling regulates cell-cell and cell-matrix adhesion (Jones et al., 2018). Phenotypically, blockade of Fgf10 in the early developing lung results in the partial disruption of the lumen within the lung bud as well as increased density of the epithelial layer. All of these changes are likely the consequences of cell rearrangements within the epithelial layer. Interestingly, no changes in the proliferation or survival of the embryonic lung epithelium was observed upon inhibition of Fgfr2b signaling indicating that at these early stages, Fgfr2b signaling mostly impacts cell adhesion.

## In Humans, Ectopic Fgf10 Expression is Associated With Tumor Progression in 10% of the Breast Cancers

In humans, FGF10 is expressed in both normal and breast cancer tissue, being detectable in 92% of the primary tumors (Theodorou et al., 2004). Supporting the paracrine nature of FGF10 signaling described during early mammary gland development FGF10 expression in normal and breast cancer tissue is limited to the stromal fibroblasts- Luminal epithelial cells of the normal human breast duct do not express FGF10 (Grigoriadis et al., 2006). However, in 10% of the breast carcinomas displaying a high epithelial/stroma ratio, FGF10 is ectopically expressed in the epithelium at high level (Theodorou et al., 2004). Interestingly, some breast carcinoma cell lines show high expression of FGF10 (Theodorou et al., 2004), supporting the possible role of autocrine FGF10 signaling in human breast cancer progression.

In addition, it was reported that FGFR2 expression is drastically increased in breast cancer (Grose and Dickson, 2005; Moffa and Ethier, 2007). *FGFR2* mutations are also associated with increased risk of breast cancer in women without history of breast cancer in their family (Hunter et al., 2007). It is likely that such high level of expression of the receptor leads to the significant activation of the FGF10/FGFR2b signaling pathway due to the large amount of FGFR2b ligands expressed in the adult mammary gland. Genetic variants near the *FGF10* locus have been identified through genome-wide association studies and their detection is considered as a risk factor for breast cancer formation (Stacey et al., 2008). It has also been reported that in the context of *FGFR2* genetic variants and breast cancer, lower FGFR2 expression is associated with increased response to estrogen (Campbell et al., 2016) and these FGFR2 variants are associated with poor prognosis (Castro et al., 2016). FGF10 stimulation of the breast cancer cell line MCF-7 [a cell line described as estrogen receptor-positive (ER^pos^)] drive the cells to a basal-like cancer phenotype with diminished dependency to estrogen associated with decreased sensitivity to treatments with anti-estrogen. Interestingly, ER^pos^ breast cancer cells display increased response to the anti-estrogen tamoxifen when treated with AZD4547 and PD173074, two well-known FGFR inhibitors. Therefore, it appears that inhibition of FGF10-FGFR2 signaling can be used therapeutically to bypass the resistance to anti-hormone therapy in the context of breast cancer treatments (Campbell et al., 2018). Patients with FGFR2-overexpressing breast tumors display poor survival when treated with lapatinib, a tyrosine kinase receptor inhibitor for EGFR and HER2 suggesting that FGFR2 signaling could be maintaining the self-renewal and differentiation capabilities of breast cancer stem cells (BCSCs) in the context of lapatinib treatment (Sridharan et al., 2019). Through its binding to FGFR2b, FGF10 triggers increased cell migration and proliferation. This is achieved via the activation of the ERK1/2 pathway, leading to an increased activity of Cyclin D2 and its downstream target E2F. Concomitantly, FGFR2b signaling leads to increased PI3K-AKT activity resulting in the inhibition of GSK3ß and the subsequent accumulation of ß-catenin culminating in upregulation of WNT signaling. Interaction of FGF10 with FGFR1 furthermore leads to the cleavage of the 55 kDa C-terminal fragment by granzyme B (GrB) and its translocation to the nucleus, where it promotes the transcription of target genes related to cell migration and proliferation (Figure 2E). Supporting this possibility that FGFR2 signaling could be maintaining the self-renewal and differentiation capabilities of breast cancer stem cells (BCSCs), FGFR2 overexpressing cells are resistant and proliferate under lapatinib selection. It has therefore been proposed that additional anti-FGFR treatment could be beneficial for breast cancer patients treated unsuccessfully with lapatinib (Azuma et al., 2011). Indeed, the use of the PI3K/mTOR inhibitor NVP-BEZ235 in association with the pan-RTK inhibitor dovitinib has been reported to be more beneficial than treatment with single inhibitors (Issa et al., 2013). In mice, efficient responses in terms of tumor growth and apoptosis are associated with the use of FGFR inhibitor in combination with either the PI3K/mTOR inhibitor or the pan-ErbB inhibitor (Issa et al., 2013). In addition, GP369 is a new therapeutic tool in our anti-FGFR arsenal. This monoclonal antibody specifically binds and inactivates the FGFR2-IIIb receptor isoform which is specifically expressed in the epithelium and which overexpression is associated with tumorigenesis (Hackenberg et al., 1991). In transplantation experiments with MFM-223 breast cancer cells (a cell line with 287 genomic copies of FGFR2), the administration of GP369 prevents tumor growth (Bai et al., 2010). Interestingly, FGF10 expression could be regulated through a non-coding antisense RNA called *FGF10-AS1*. Low level of *FGF10-AS1* is associated with triple negative breast cancer (Fan et al., 2019).

## In Conclusion

Developmental biology and cancer research are revealing complementary aspects of FGF signaling in both the normal biology and the pathological processes of the mammary glands. The characterization of the molecular and cellular basis of mammary placode induction allows to get a better insight in the control of proliferation, migration and differentiation. These developmental processes are often simultaneously mis-regulated during breast cancer progression and metastasis. Understanding these developmental processes may therefore offer potential novel therapeutic targets for breast cancer treatment.

## Author Contributions

SR and SB wrote the review and made the illustrations. ChaC and CheC contributed to the revisions and made the updated figures. All authors contributed to the article and approved the submitted version.

## Conflict of Interest

The authors declare that the research was conducted in the absence of any commercial or financial relationships that could be construed as a potential conflict of interest.
